# Fast and Automatic Activation of an Abstract Representation of Money in the Human Ventral Visual Pathway

**DOI:** 10.1371/journal.pone.0028229

**Published:** 2011-11-30

**Authors:** Catherine Tallon-Baudry, Florent Meyniel, Sacha Bourgeois-Gironde

**Affiliations:** 1 Centre de Recherche de l'Institut Cerveau-Moelle, Universite Pierre et Marie Curie (UPMC), Centre National de la Recherche Scientifique (CNRS), Institut National de la Santé et de la Recherche Médicale (INSERM), Paris, France; 2 Institut Jean-Nicod, Ecole Normale Supérieure (ENS), Paris, France; French National Centre for Scientific Research, France

## Abstract

Money, when used as an incentive, activates the same neural circuits as rewards associated with physiological needs. However, unlike physiological rewards, monetary stimuli are cultural artifacts: how are monetary stimuli identified in the first place? How and when does the brain identify a valid coin, *i.e.* a disc of metal that is, by social agreement, endowed with monetary properties? We took advantage of the changes in the Euro area in 2002 to compare neural responses to valid coins (Euros, Australian Dollars) with neural responses to invalid coins that have lost all monetary properties (French Francs, Finnish Marks). We show in magneto-encephalographic recordings, that the ventral visual pathway automatically distinguishes between valid and invalid coins, within only ∼150 ms. This automatic categorization operates as well on coins subjects were familiar with as on unfamiliar coins. No difference between neural responses to scrambled controls could be detected. These results could suggest the existence of a generic, all-purpose neural representation of money that is independent of experience. This finding is reminiscent of a central assumption in economics, money fungibility, or the fact that a unit of money is substitutable to another. From a neural point of view, our findings may indicate that the ventral visual pathway, a system previously thought to analyze visual features such as shape or color and to be influenced by daily experience, could also able to use conceptual attributes such as monetary validity to categorize familiar as well as unfamiliar visual objects. The symbolic abilities of the posterior fusiform region suggested here could constitute an efficient neural substrate to deal with culturally defined symbols, independently of experience, which probably fostered money's cultural emergence and success.

## Introduction

How does the brain react to money? Money is a powerful incentive, that activates the same neural circuits than rewards associated with physiological needs, such as food or sex [1], [2], [3], [4], [5], despite the fact that the status of money as a reward is not innate but has been acquired by experience. However, for a monetary incentive to influence brain activity and behavior, it first has to be identified as being money at the neural level. In other words, the basic neural processing step of money recognition has to be performed before any economic behavior can take place. This is analogous to the notion that a prerequisite for social behavior is to be able to distinguish a face from another visual object. The objective here is therefore to understand how and when the brain does assign the label “money” to a visual input.

Before answering this question, one has to refine money's definition. If we restrict this category to one of its most typical instances, coins [6], what differentiates a coin – i.e., valid money – from a disc of metal of similar visual aspect is that a coin can be exchanged for goods and services. Money validity therefore relies on a social agreement: a coin is endowed with monetary properties if and only if everyone agrees it can be used as money. In that sense money is a symbol [7], and it has validity much in the same way as words have meaning [8]. The nature of the category “coin” is therefore very different from the nature of categories like birds or faces that are mostly based on visual similarity. This leaves the issue of where and when in the brain a coin is recognized as money quite open.

We were also interested in the nature of money representation at the neural level. Anthropologists [9], sociologists [10] and psychologists [6] have suggested the existence of a polymorphous representation of money. For instance, money won at a lottery has a different nature than money earned by working for long hours. However, a central principle in economics is that money is fungible. In other words, the different instantiations of money are fundamentally exchangeable: a dollar bill should, according to theory, be equivalent to a dollar coin. This latter view would imply the existence of a single, general-purpose mental representation of money, while the polymorphous account would predict that money representation is dependent on personal experience.

**Figure 1:**
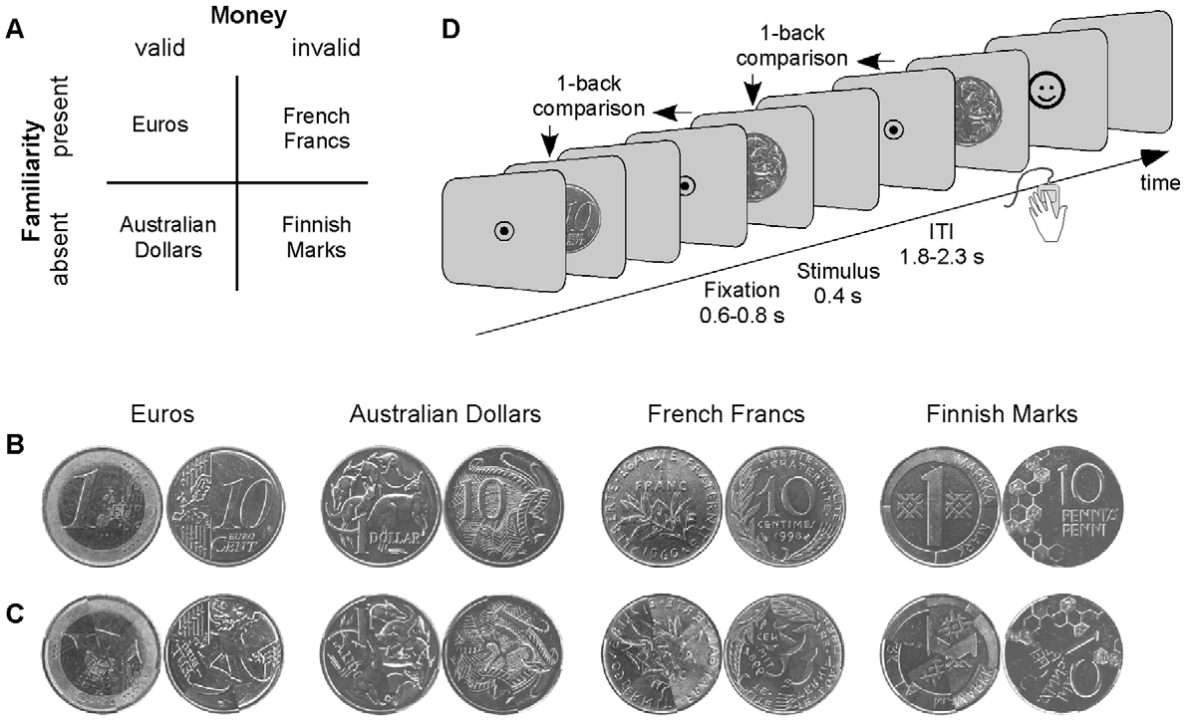
Paradigm. **A.** Experimental design: each coin presented could be valid or invalid, familiar or unfamiliar for the French subjects who participated in the experiment. Note that French Francs and Finnish Marks were replaced by Euros in 2002 and are no longer in use. **B.** Stimuli: Unit of currency and 10 cent coins, for Euros, Australian Dollars, French Francs and Finnish Marks. **C.** Scrambled controls. **D.** 1-back task: subjects had to press a button whenever two identical stimuli were presented in a row.

To investigate these issues, we manipulated experimentally two factors: validity, or whether a disc of metal is endowed with monetary properties or not, and familiarity, or the amount of prior experience the subject has had with a given type of coins. We took advantage of the change made in 2002 in the Euro zone: local currencies were replaced by Euros. In particular, the French Francs and Finnish Marks used in our study retained their visual appearance, but lost their purchasing power and became what we termed invalid coins. To identify when and where money is detected in the brain, we compared the neural responses to these invalid coins with responses to Euros and Australian Dollars, that are currently in use and therefore valid. We deliberately used coins that were familiar to the French subjects who participated in the experiment (French Francs and Euros) as well as unfamiliar ones (Australian Dollars and Finnish Marks): if there is a generic neural representation of the category “money”, independently of personal experience, then similar responses should be observed for familiar and unfamiliar coins. Each coin could therefore be either valid or invalid, and familiar and unfamiliar (Figure 1A). To vary perceptual inputs, we used two different coins for each currency, normalized to the same size and luminance (Figure 1B). To control for the influence of potential low-level confounding factors, we created scrambled counterparts of each stimulus (figure 1C) and presented the scrambled stimuli randomly intermixed with the coins. These additional scrambled stimuli controlled for potential low-level influences, such as spatial frequency content, between coin types. Each stimulus could therefore be classified as a coin or a scrambled control (factor Object Type), as endowed with monetary validity or not (factor Validity), and as familiar or not (factor Familiarity).

We first ascertained that each participant never had any experience with Australian Dollars nor with Finnish Marks, and was fully aware of the current monetary status of coins. Each coin was visually presented in a slideshow, along with a written sentence describing its value (unit of currency or 10 cents), its country of origin, whether it is currently in circulation or whether it was in use until 2002. Note that the terms “valid” and “invalid” were not used in the subjects' instructions, neither in the slideshow nor later in the experiment. Each coin was then displayed along with a multiple-choice questionnaire. Subjects had to click on the correct answers: Unit or 10 Cents, in use or no longer in use, from France, Finland, Euro area or Australia. The procedure was repeated until subjects could accurately characterize all coins, a performance reached within a few minutes. Subjects were therefore fully aware of basic monetary facts about all coins before the beginning of the recordings. We then recorded the magneto-encephalographic (MEG) neural responses to the different stimuli. Because we were interested in how stimuli are automatically categorized as being valid money or not, even when this dimension is not relevant, money validity was incidental to the task: subjects had to press a button whenever the same stimulus, be it a coin or a scrambled control, was presented twice (Figure 1D).

## Results

### Behavior

The 16 participants performed correctly the one-back task (mean performance: 95.3%±sem 0.79; mean reaction times 551.5 ms±19.9, see Table 1 and Figure S1 for details), confirming they remained attentive to the low-level visual properties of the stimuli throughout the experiment. The factors Object Type, Validity and Familiarity were incidental to the one-back detection task and had only mild, non-significant influences on subjects' behavior: there was a non significant trend toward a faster detection of repeated coins compared to repeated scrambled stimuli (main effect of Object Type on reaction times, F(1,15) = 2.74, p = 0.12; all other F(1,15)<1.57, p>0.22 for main effects and interactions). In addition, the repetition of unfamiliar stimuli tended to be slightly better detected than the repetition of familiar stimuli, although this effect did not reach significance (main effect of Familiarity on percent correct: F(1,15) = 3.5, p = 0.081; all other F(1,15)<1.64, p>0.2 for main effects and interactions). Behavioral results therefore suggest that low-level visual properties, that are relevant to detect a repetition, were relatively similar in the different experimental conditions. Any influence of the experimental factors on neural activity is therefore more likely to be due to automatic categorization processes rather than to low-level differences between stimuli.

**Table 1:** Behavioral data (mean ± standard error of the mean).

	Coins				Scrambled controls			
	Euros	Francs	Dollars	Marks	Euros	Francs	Dollars	Marks
RT (ms)	542.4±24.9	541.2±28.7	542.7±22.4	543.9±24.2	568.7±20.3	578.2±28.8	547.5±21.3	547.1±23.4
% correct	91.8±2.4	96.5±1.1	96.1±1.0	95.7±1.1	94.1±2.0	94.9±1.2	96.5±1.0	96.9±1.6

### MEG responses

**Figure 2:**
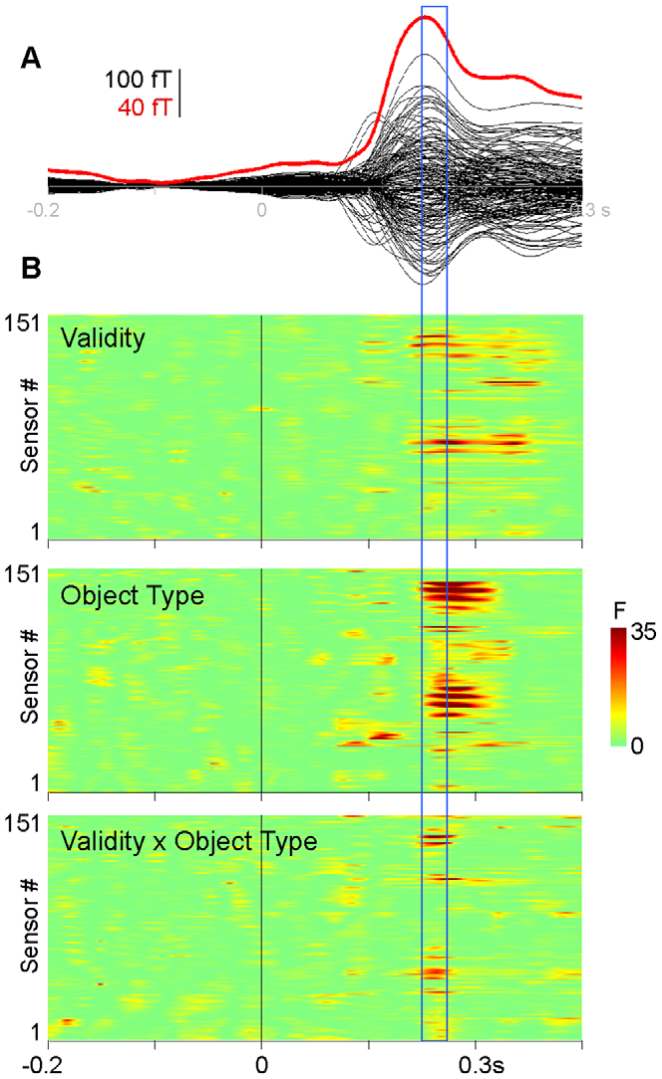
Time-courses. **A.** Superimposed evoked fields at the 151 channels (black) and root mean square across sensors (red), grand average across subjects and conditions. **B.** Statistical plots showing the F-value (color code) of the effect of monetary Validity (top), Object Type (middle) and their interaction (bottom), depending on time (x axis) and sensors (y axis). Both Validity, and the interaction Validity x Object Type, affect event-related fields between 150 and 175 ms (blue box).

To test whether monetary validity had an impact on neural activity, we analyzed the evoked MEG neural responses to non-repeated stimuli. We first ran a 3-way ANOVA with the factors Object Type (coin or scramble), monetary Validity, and Familiarity on all sensors, searching for a time-window showing an interaction between Object Type by monetary Validity and/or a main effect of monetary Validity. Both the interaction between monetary Validity and Object Type and the main effect of monetary Validity occurred surprisingly early, between 150 and 175 ms (figure 2). A prominent effect of Object Type could also be seen in this time-window. It therefore seems that the visual system can distinguish between valid and invalid coins in the 150–175 ms range. In other words, the human visual brain would be able to distinguish between images that are visually similar, but endowed with distinct monetary properties, at surprisingly early latencies.

**Figure 3:**
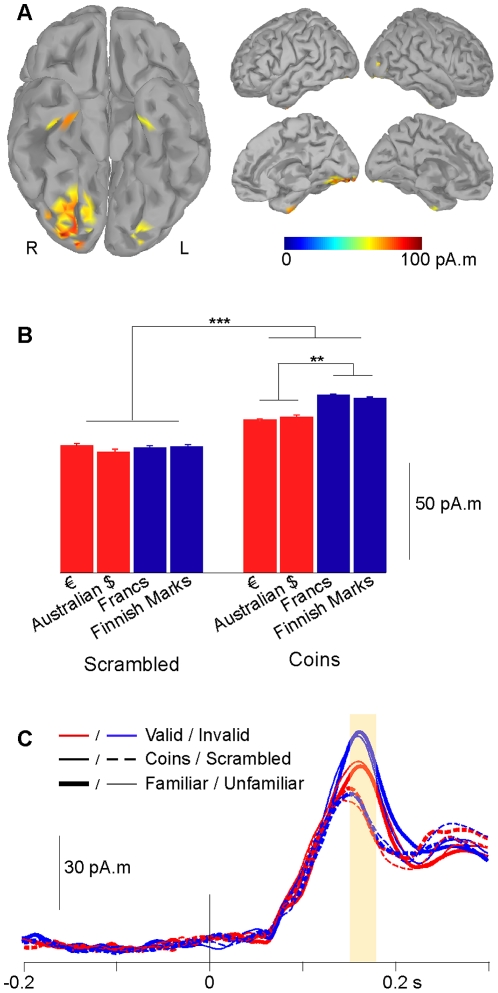
Estimated neural sources of the mean 150–175 ms activity. **A.** The 60% top-most responsive regions (yellow/red scale) are all located in the ventral visual pathway, in the posterior part of the right fusiform and lingual gyri. R: right, L: left. **B.** Mean 150–175 ms activity in the right posterior fusiform and lingual region, in response to scrambled controls (left) and coins (right), valid (red) or invalid (blue). Responses to controls are smaller than to coins, and responses to valid coins are smaller than responses to invalid coins. ***: p<0.001; **: p<0.01 **C.** Time-courses of neural responses in the posterior fusiform and lingual region. In this region the dissociation between coins (solid lines) and scrambled controls (dotted lines) is quickly followed by a dissociation between valid (red solid lines) and invalid (blue solid lines) coins, corresponding to the significant interaction between Object Type and Validity. Familiarity with the coins does not affect the responses (thin vs. thick lines). The yellow box indicates the 150–175 ms time range.

To investigate this possibility further, we computed the neural sources underlying MEG data in the 150–175 msec time-window, using a minimum-norm estimate (figure 3A). Sources were estimated separately for each condition and each subject, and averaged across conditions and subjects to determine the most responsive areas, independently from the factors of interest. In the 150–175 ms time-window, the most active regions, across subjects and conditions, were located along the right posterior fusiform and lingual gyri. Averaging neural activity per condition in this region over the 150–175 ms time window (figure 3B) revealed that scrambled stimuli give rise to a much smaller response than coins (main effect of object type, F(1,15) = 24.6 p<0.0002). Importantly, in the same latency range, neural responses in the vicinity of the fusiform gyrus discriminated between valid and invalid coins (main effect of validity, F(1,15) = 8.7 p<0.01; interaction between object type and validity, F(1,15) = 8.99 p<0.01). No other main effect nor interaction reached significance (all F(1,15)<1.4, p>0.25).

One might expect that familiar coins are more readily discriminated than unfamiliar ones: behaviorally, familiarity seems relevant – for instance subjects tend to behave as if familiar coins had a larger purchasing power [11]. However familiarity did not affect neural activity in this time range (main effect of familiarity, all interactions involving familiarity: all F(1,15)<1.4, all p>0.25). The effect of validity was present for familiar coins (Euros vs. French Francs, paired t-test, t(15) =  −2.36, p = 0.032) as well as for unfamiliar coins (Australian Dollars vs. Finnish Marks, paired t-test, t(15) =  −2.37, p = 0.032). The size of the validity effect (difference between valid and invalid coins) was similar in male and female participants (males, 11.1 pA.m; females, 10.75 pA.m; unpaired ttest p>0.96). Because the number of female participants was small (n = 4), gender differences may not be detected here. Altogether, the pattern of results suggests that our brain assigns the socially-defined label “money” to valid coins by a process that is not sensitive to daily experience.

We then extracted the time course of activity in the region of interest (figure 3C). For both coins and scrambled controls, activity rises at about 80 ms. Around 140 ms responses to coins and responses to scrambled stimuli begin to differ, and almost at the same time, neural responses to valid and to invalid coins diverge. In this region, it therefore appears that neural activity distinguishes between valid and invalid coins almost at the same time that it differentiates objects from scrambled controls.

To what extent does the validity effect depend on coins numerical value? Are there different responses to 10 cents coins and unit coins? There was no significant influence of numerical value in the posterior fusiform and lingual region in the 150–175 ms time window (two-way ANOVA, main effect of Object Type F(1,15) = 22.24, p<0.0003, main effect of Numerical Value F(1,15) = 3.38, p = 0.08, interaction F(1,15) = 2.09, p>0.16). However, in a later time-window, between 175 and 200 ms, numerical value influenced neural responses in this region, with a significant interaction between Object Type and Numerical Value (F(1,15) = 12.03, p = 0.0034; main effect of Numerical Value F(1,15) = 0.18, p = 0.68; main effect of Object Type F(1,15) = 19,7 p<0.0005). The differential activity between valid and invalid coins in the posterior fusiform and lingual region therefore appears before money's numerical value affects neural activity.

## Discussion

The ventral visual system, known to be involved in the perceptual analysis of objects' shapes [12], [13], [14], discriminates valid from invalid coins at early latencies, between 150 and 175 ms. This discrimination does not rely on low-level visual attributes, since no differential effects can be seen in scrambled controls. Rather, the socially-defined concept of monetary validity seems to be incorporated at early latencies in visual signals, even when this concept is not relevant for the task at hand. The mechanism uncovered here seems to operate as well on coins subjects have repeatedly used as on coins subjects have only recently learnt about. A parsimonious interpretation of these findings is the existence of a neural representation of a generic, use-independent category “money” in the ventral visual pathway, that is automatically activated.

### The nature of money representation in the ventral visual pathway

Familiar as well as non familiar coins were readily classified as valid money in the ventral visual pathway. The fact that valid coins from different currencies gave rise to the same type of neural response indicates the presence of a categorical process. Indeed, the hallmark of categorization is that different exemplars of the same category should elicit similar responses despite some variations in sensory input [15]: for instance, the category “dog” is composed of exemplars that are visually as different as a greyhound and a Pekingese dog. In this region and at this early processing stage, the neural representation of money therefore seems to represent the *category* money: different instances of money elicit the same type of responses. Because familiar and unfamiliar coins are readily categorized in a similar way, the neural representation of money that pre-existed in each subject before the recordings must have been generic and abstract enough to accommodate new instances of money. This finding may appear to lend support to a central assumption in economics, fungibility, or the fact that any unit of money is substitutable for another. However, it is important to note that the representation of money we describe here is, from an economic point of view, a rather coarse one. Indeed, it is independent of the coins numerical value, a property that appears to be analyzed only later in the brain.

It could be argued that the distinction made between valid and invalid coins in the ventral pathway around 150 ms does not reflect monetary validity, but either a low-level confounding factor or a high-level cognitive function such as attention. To control for low-level factors we used two exemplars per category, and designed scrambled stimuli, that do not show the effect. To control for high-level cognitive confounds we minimized explicit cognitive demands by using a one-back task, but this does not prevent the automatic recruitment of high-level cognitive functions. The automatic triggering of a cognitive function by a stimulus is all the more likely that this particular function has been associated to this particular stimulus in the past. In our experiment, the difference between valid and invalid coins was seen for familiar coins, that subjects have manipulated and experienced daily in their full economic dimension, but also for unfamiliar coins, that subjects saw for the first time on the day of the recordings. It seems therefore unlikely that the automatic recruitment of a high-level cognitive function can account for our results.

Money is not only a commodity, it is also a powerful incentive and often used as a reward, in everyday life as well as in decision-making paradigms [16]. In our experiment, money was not used as a reward and participants received a fixed monetary indemnity, unrelated to performance or to the stimuli presented. However, the incidental categorization into valid or invalid money might nevertheless automatically elicit reward-related processes. It is known that visual processing can be modulated by the recent reward history of the stimulus [17], [18], [19]. To interpret the activity seen here in the ventral visual pathway as reflecting reward value, one would have to assume that reward processes can be automatically triggered independently from experience. Indeed, in the case of unfamiliar coins, subjects never had any direct experience of the stimuli as rewarding, but activity in the ventral pathway was nevertheless affected by monetary validity. In addition, evidence for reward processing is usually obtained by comparing large vs. small rewards. Here, coins numerical value appears to be analyzed only 25 ms after the stimulus has been identified as valid money. The analysis of coins numerical value was automatic and incidental to the task, in line with recent evidence for automatic valuation neural processes [20]. This effect nevertheless remains difficult to interpret further since monetary value (10 cents<1 unit) and numerical value (10>1) were going in opposite directions. Altogether, our results suggest that coins were first categorized as valid or not, and then valuated, each of these processing steps potentially including some reward-related components. It is the first step, the fast and coarse categorization of money, independently of the amount of money presented and of daily experience with the coins, that is the most surprising one.

### Conceptual properties in the posterior visual pathway?

From a neural point of view, the results are unexpected for two reasons: first, if the visual regions involved here are known to be involved in object categorization, they are also deeply shaped by experience [21], [22], [23]. One would therefore have expected a strong influence of familiarity in those areas. Second, money is a symbol, not a visual property: subjects had to be told about the monetary validity of non familiar currencies. It is well known that the categorization of natural objects such as faces can take place within ∼150 ms in the human ventral visual pathway [24]. However, the category “faces” is defined by objects that share strong visual similarities (eyes, nose and mouth, precisely organized in space). For objects whose meaning is defined by social agreement, such as words, the process usually takes much longer. For instance, categorizing a letter string as a valid word (as opposed to a pseudoword such as “sapon”) usually takes at least 300 ms [25]. In the present experiment, discs of metal are categorized as valid money or not within 150–175 ms, a speed of processing that would be similar to that of natural categories defined by visual properties despite the fact that money is defined by social agreement. Our results could therefore suggest an amazing ability of the human ventral visual system at dealing with symbolic processing on the basis of knowledge rather than experience. Since no effect was detected for scrambled controls, we attribute the observed difference between valid and invalid coins to the conceptual factor of monetary validity rather than to low-level visual feature similarities. However, series of experiments in various countries and using different coins would be needed to definitively validate this interpretation of our findings.

Is money the only conceptual, experience-independent, category treated at such an early level in the visual system? It is too early to provide a definitive answer, but at least another conceptual category (living vs. non living items) shows some experience-independent neural organization [26]. It seems unlikely that the pattern of activity we observe arises from a specialized functional module, dedicated to money *per se*, because money is a much too recent invention (∼3000 years) to have influenced brain evolution [27]. Rather, the ability to categorize money is probably rooted in evolutionary ancient abilities of the ventral visual system to process symbols. In the case of money processing, the necessary neural machinery seems to be already present in monkeys since they can learn to use coins [28]. More generally, our results suggests that as for other cultural inventions such as reading or arithmetic [29], cultural abilities do not necessarily arise from distributed, high-level flexible neural mechanisms but can take place in dedicated cortical territories that were originally devoted to other, more ecological purposes. Whatever the primitive mechanism money perception is rooted in, our results indicate that money, that is defined by social agreement, is categorized in the ventral visual pathway as fast as natural, non symbolic objects defined by their visual properties. This surprising neural fluency at dealing with coins probably participated to money's worldwide success.

## Materials and Methods

### Ethics statement

All subjects gave their written informed consent prior the experiment. All procedures were approved by the local ethics committee (Comité Consultatif de Protection des Personnes dans la Recherche Biomédicale, Hôpital de la Pitié-Salpêtrière, Paris, France).

### Subjects

Sixteen right-handed subjects (4 females) with normal or corrected to normal vision participated in the experiment. All subjects were old enough (mean age 33.15 years±0.8 sem, range 30–39 years) to have managed a budget in Francs, since they were on average 27 years old in 2002, when Euros replaced Francs. Subjects were paid 45 Euros for their participation.

### Stimuli

Real coins were photographed under natural light conditions, scaled to the same size and converted to black and white. Luminance (mean and standard deviation) was equated across pictures. Control stimuli were created by dividing each coin in 8 pie-slices and shuffling the slices. Stimuli covered the central 2 degrees of the visual field at a viewing distance of 85 cm. All stimuli were presented via a mirror system on a grey background (luminance: 26.9 cd per m2) at the center of a back projection screen, using a calibrated Mitsubishi X120 projector (resolution: 1024×768 pixels, refresh rate: 60 Hz) located outside the shielded recording room. The luminosity of the recording room was controlled as well as the luminance of the grey background of the projection screen using a Konica Minolta LS-100 luminance meter.

### Task, Procedure and Recordings

The experiment was divided in 8 blocks of 96 trials. Trials within a block were presented in a pseudo-randomized order different for each subject. Each block consisted in 6 presentations of each coin exemplar (12 presentations per currency) or scramble control, with one immediate repetition that was not included in MEG data analysis. Subjects were instructed to make an unspeeded button-press with their right hand whenever a picture was preceded by exactly the same picture. As illustrated in figure 1D, each trial began with the presentation of a central fixation disc presented for 0.6 to 0.8 second, followed by the stimulus (0.4 s), and a blank screen (inter-trial interval, 1.8 to 2.3 s). If the subject pressed a button during the inter-trial interval, a positive (green smiley) or negative (red smiley) feed-back was presented for 0.2 s followed by the blank intertrial screen for 1.8 to 2.3 s. If the subject failed to respond after a repetition, the negative feedback was delivered after 2.3 s, followed by the 1.8–2.3 s inter-trial interval. One training block was performed before recording.

Continuous magneto-encephalographic signals were collected using a whole-head MEG system with 151 axial gradiometers (CTF Systems, Port Coquitlam, BC, Canada) at a sampling rate of 1250 Hz and low-pass filtered online at 300 Hz. Head localization with respect to the MEG sensor array was measured at the beginning of each recording block using marker coils that were placed at the cardinal points of the head (nasion, left and right ear). Vertical and horizontal electrooculogram (EOG) signals were simultaneously collected.

### Data Analysis

Data analysis was performed using in-house software (http://cogimage.dsi.cnrs.fr/logiciels/), and additional programs developed in MATLAB (The MathWorks, Natick, MA). All time samples were corrected with respect to the refresh delay of the projector (+25 ms). Trials contaminated with eyes movements (rejection threshold: 1.0 degree of visual angle from fixation), eye blinks or muscular artifacts were rejected offline upon visual inspection of their unfiltered EOG and MEG traces. On average, 59.6 trials±3.2 sem per condition were averaged. To calculate event-related magnetic fields (ERFs), epochs from 200 ms pre- to 300 ms post-stimulus onset were averaged for each condition and participant, baseline corrected using the 200 ms preceding stimulus onset and low-pass filtered at 30 Hz. ERFs were computed separately for each subject and condition and entered into repeated-measure ANOVAs with Object Type (coin or scrambled control), monetary Validity (present or absent) and Familiarity (present or absent) as factors.

### Source localization

Cortical current density mapping was obtained using a distributed model consisting of 15.000 current dipoles in each subject and in each condition. Dipole locations and orientations were constrained to the cortical mantle of a generic brain model built from the standard brain of the Montreal Neurological Institute using the BrainVISA software (http://brainvisa.info). Source localization and surface visualization was performed with BrainStorm [30], which is documented and freely available for download online under the GNU general public license (http://neuroimage.usc.edu/brainstorm). Cortical current maps were computed from the MEG time series using a linear inverse estimator (weighted minimum norm current estimate), separately for each condition.

## Supporting Information

Figure S1 Behavior. A. Accuracy. B. Reaction times. Results for valid coins are shown in red, for invalid coins in blue. There was no significant main effect nor interaction for the factors of interest (Object Type, Validity, Familiarity) on the performance in the one-back task, that relies mostly on low-level visual information.
(TIF)
