# Supplementary figures and images for: Fast and Automatic Activation of an Abstract Representation of Money in the Human Ventral Visual Pathway
Source: PLoS One. 2011 Nov 30;6(11):e28229. doi: 10.1371/journal.pone.0028229 (PMC3227657; doi:10.1371/journal.pone.0028229)

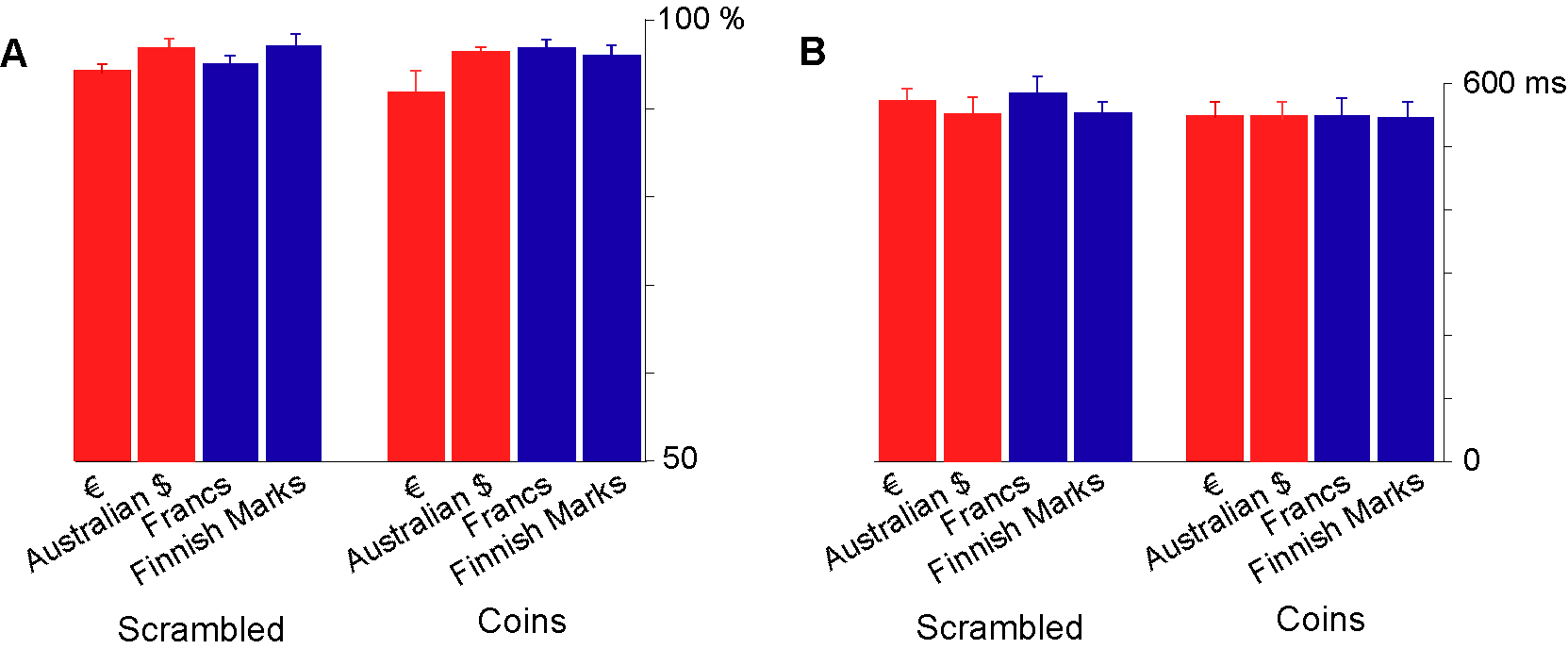

Supplement: Figure S1 — Behavior. A. Accuracy. B. Reaction times. Results for valid coins are shown in red, for invalid coins in blue. There was no significant main effect nor interaction for the factors of interest (Object Type, Validity, Familiarity) on the performance in the one-back task, that relies mostly on low-level visual information. (TIF) [file pone.0028229.s001.tif]
